# Supporting decision making on public health and social measures in response to COVID-19- The PHSM calibration tool: Dominic Cocciolone

**DOI:** 10.1093/eurpub/ckac129.536

**Published:** 2022-10-25

**Authors:** D Cocciolone, K King, A Latta, S Lindmark, L Owen, I Perehinets, C Roman, J Sane, T Schmidt, C Wippel

**Affiliations:** Country Health Emergency Preparedness and IHR, WHO Regional Office for Europe, Copenhagen, Denmark

## Abstract

Public health and social measures (PHSM) are preventative measures taken by individuals, communities and government institutions at national and local levels to prevent and reduce transmission of an infectious disease - in this instance SARS-CoV-2. The decision to introduce, adapt or lift PHSM should be based primarily on a situational assessment of the intensity of transmission of SARS-CoV-2 and the capacity of the health system to respond to subsequent increases in hospital admissions, but must also consider the effects these measures may have on the general welfare of society and individuals. The WHO Regional Office for Europe developed an online public health and social measures (PHSM) calibration tool to assist Member States in decision-making relating to PHSM implementation during the COVID-19 pandemic. The tool, designed to be used primarily by policy-makers in national and local government authorities, provides guidance based on a situational-level assessment framework that is determined by the level of community transmission and the overall capacity of health systems and public health services within a country or region to respond. By using a combination of country-reported and user-input data, the tool automatically generates a situational assessment and corresponding PHSM guidance for users, summarized in a downloadable report.

**Reference:**

https://phsm.euro.who.int/calibrationTool

